# The Optimal Amount of PAMAM G3 Dendrimer in Polyurethane Matrices Makes Them a Promising Tool for Controlled Drug Release

**DOI:** 10.3390/polym18010135

**Published:** 2026-01-01

**Authors:** Magdalena Zaręba, Magdalena Zuzanna Twardowska, Paweł Błoniarz, Jaromir B. Lechowicz, Jakub Czechowicz, Dawid Łysik, Magdalena Rzepna, Łukasz Stanisław Uram

**Affiliations:** 1 Faculty of Chemistry, Rzeszów University of Technology, 6 Powstańców Warszawy Ave, 35-959 Rzeszów, Poland; magzar@prz.edu.pl (M.Z.); d625@stud.prz.edu.pl (M.Z.T.); bloniarz@prz.edu.pl (P.B.); jlechow@prz.edu.pl (J.B.L.); 176554@stud.prz.edu.pl (J.C.); 2 Institute of Biomedical Engineering, Bialystok University of Technology, Wiejska 45C, 15-351 Bialystok, Poland; d.lysik@pb.edu.pl; 3 Centre for Radiation Research and Technology, Institute of Nuclear Chemistry and Technology, Dorodna 16, 03-195 Warsaw, Poland; m.rzepna@ichtj.waw.pl

**Keywords:** PAMAM dendrimer, polymer matrix, doxorubicin, encapsulation, release profile

## Abstract

Systemic anticancer therapy causes a number of side effects; therefore, local drug release devices may play an important role in this area. In this study, we developed polyurethane-dendrimer foams containing different amounts of third-generation poly (amidoamine) dendrimers (PAMAM G3) to evaluate their ability to encapsulate and release the model anticancer drug doxorubicin (DOX), as well as their biocompatibility and effectiveness against normal and cancer cells in vitro. PU–PAMAM foams containing 10–50 wt% PAMAM G3 were prepared using glycerin-based polyether polyol and castor oil as co-components. Structural and rheological analyses revealed that foams containing up to 20 wt% PAMAM G3 exhibited a well-developed porous structure, while higher dendrimer loadings (≥30 wt%) led to irregular cell shapes, pore coalescence, and thinning of cell walls, and indicated a gradual loss of structural integrity. Rheological creep–recovery measurements confirmed the structural findings: moderate PAMAM G3 incorporation (≤20 wt%) increased both the instantaneous and delayed elastic modulus (E_1_ ≈ 130–140 kPa; E_2_ ≈ 80 kPa) and enhanced elastic recovery, reflecting improved cross-link density and foam stability. Higher dendrimer contents (30–50 wt%) caused a decline in these parameters and higher viscoelastic compliance, indicating a softer, less stable structure. The DOX loading capacity and encapsulation efficiency increased with PAMAM G3 content, reaching maximum values of 35% and 51% for 30–40 wt% PAMAM G3, respectively. However, the most sustained DOX release profiles were observed for matrices containing 20 wt% PAMAM G3. Analysis of cumulative release and kinetic modeling revealed a transition from diffusion-controlled release at low PAMAM contents to burst-dominated release at higher dendrimer loadings. Importantly, matrices containing 10–20 wt% PAMAM G3 also indicated selective anticancer action against squamous cell carcinoma (SCC-15) compared to non-cancerous human keratinocytes (HaCaT). Moreover, the DOX they released effectively destroyed cancer cells. Overall, PU–PAMAM foams containing 10–20 wt% PAMAM G3 provide the most balanced combination of structural stability, controlled drug release, and cytocompatibility. These materials therefore represent a promising platform as passive carriers in drug delivery systems (DDSs), such as local implants, anticancer patches, or bioactive wound dressings.

## 1. Introduction

Currently, there is significant and constantly growing demand for novel drug delivery systems (DDSs). The primary goal is to utilize appropriate carriers to enhance drug stability, extend their release time and deliver the drug to the target site [1,2,3]. Among many DDSs, transdermal and local delivery systems are one of the most promising tools for delivering active substances, because they offer non-invasive routes and minimize numerous side effects [4,5,6]. One form of DDS comprises polymer matrices capable of immobilizing drugs and releasing them in a controlled and prolonged manner at the target site [7,8,9]. Such systems are used as transdermal patches, implants or injectable scaffolds [10,11,12,13,14]. Polyurethane (PU)-based matrices have gained attention due to their tunable porosity, biocompatibility and ease of chemical modification, making them excellent candidates for hybrid polymer–nanocarrier systems [15,16,17].

One of the most intensively studied vehicles for drugs are poly (amidoamine) dendrimers (PAMAMs), also used in targeted and transdermal transport [18,19,20]. PAMAMs are characterized by a highly branched, tree-like structure with a central core and numerous repeating surface groups on the molecule surface [21,22]. They have a well-defined, monodisperse character, which means that they have a precise, spherical shape and predictable molecular weight [23,24]. The branched structure of PAMAM dendrimers, their polyvalence, and free spaces between dendrons as well as satisfactory biocompatibility and bioavailability give these nanoparticle drug carriers useful properties [25]. Drug binding to the dendrimer carrier can occur via surface conjugation or encapsulation inside the dendrimers’ cavities. Encapsulation of hydrophobic substances in the cavities of dendrimers protects them and allows for their release in a controlled manner [20,26]. Different PAMAM dendrimer generations differ in the shape and size of their particles, functionality, and the size of cavities, their accessibility, and toxicity due to differences in cationic surface charge. A distinction is made between lower- and higher-generation dendrimers, and their use as drug carriers is related to this division. PAMAM G3 can be considered as a bridge between lower and higher generations of dendrimers. G2-generation dendrimers do not possess a well-formed spherical structure or sufficiently large cavities for encapsulation. In contrast, higher generations of PAMAMs than G3 have larger cavities but also greater cytotoxicity and a higher cost of synthesis. PAMAM dendrimers exhibit increased cytotoxicity against both normal and cancer cells with increasing generation due to the higher density of terminal primary amine groups. These terminal amines interact electrostatically with cellular membranes, leading to membrane destabilization and disruption. Studies have demonstrated that PAMAM dendrimers possess internal free volume that increases with dendrimer generation. These dynamic voids, created by flexible and constantly rearranging branches, allow higher-generation PAMAM dendrimers to encapsulate a greater number of small molecules. This structural behavior explains the increased capacity of higher-generation dendrimers, but also their stronger negative impact on membranes, limiting their direct biomedical applications [27,28,29,30]. Therefore, the PAMAM G3 dendrimer offers an optimal balance between cavity size and cytotoxicity.

Although PAMAM dendrimers are widely studied as drug carriers, the incorporation of PAMAM G3 into the structure of a porous foam intended for drug immobilization is a new solution. The first method for obtaining such foams was described in our patent PL247079B1 [31], where 50% by weight of PAMAM G3 dendrimer was used. With an average diameter of approximately 3.6 nm [32], PAMAM G3 is sufficient to enable efficient encapsulation of molecule drugs such as DOX [33,34], while maintaining good miscibility with the polyol component. Studies on the anticancer activity of PAMAM–DOX complexes have demonstrated improved cellular uptake and enhanced cytotoxicity against cancer cells via electrostatic or encapsulation-based mechanisms [35,36]. However, their biomedical application is limited by dendrimer-related cytotoxicity and rapid release, as well as insufficient control over drug diffusion [37]. Furthermore, most previous studies have focused on free PAMAM–DOX complexes rather than on solid matrices modified with dendrimers [35,36,37,38,39,40]. The present study fills this gap by investigating how the quantitative incorporation of PAMAM G3 into polyurethane foams affects the DOX binding efficiency and controls its release.

The in vitro biological studies on human immortalized keratinocytes (HaCaTs) and the human squamous carcinoma SCC-15 cell line indicated that polyurethane matrices containing 50% PAMAM G3 released the anticancer drug doxorubicin [41]. Unfortunately, the cytotoxicity of the studied matrices was too high. Moreover, previous studies focused on systems with high dendrimer loading (e.g., 50 wt%), without systematically assessing the effect of varying the PAMAM fraction on the foam structure, viscoelastic properties and drug release capacity. Furthermore, the PAMAM dendrimer is the most expensive ingredient among all those used to synthesize the matrices; therefore, determining its minimum effective amount that ensures both structural stability and therapeutic functionality is crucial. The main goal of this work was to determine the optimal PAMAM G3 content in polyurethane matrices to achieve a balance between matrix porosity, viscoelastic stability, degree of cytotoxicity, and the efficiency of doxorubicin (DOX) immobilization and release. For this purpose, PU–PAMAM foams containing between 10–50 wt% dendrimer were synthesized and characterized in terms of structure, porosity, and rheological properties, as well as doxorubicin loading capacity and release. This work also provides new insights into the influence of the quantitative composition of PU–PAMAM systems on their biological properties, particularly significant in local anticancer therapy.

In polymer-based drug delivery systems, increasing the content of functional additives often improves selected parameters, such as drug loading capacity or release rate, but may simultaneously compromise other key properties, including structural stability or biocompatibility. Therefore, the concept of an “optimal” composition should not be equated with the maximum value of a single parameter. In this work, the optimal PAMAM G3 content is defined as a balanced compromise between structural integrity, viscoelastic stability, drug loading capacity, release kinetics, and cytotoxicity, providing the most favorable overall performance rather than the highest value of individual metrics.

## 2. Materials and Methods

### 2.1. Materials

The polyoxyalkylene triol (Rokopol^®^G441) was supplied by PCC Rokita (Brzeg Dolny, Poland). 4,4′-Diphenylmethane diisocyanate (mixture of di- and tri-isocyanates) for synthesis, (pMDI, Merck Life Science, Darmstadt, Germany); Triethylamine (TEA, 99.5%, Chempur, Piekary Śląskie, Poland); Silicone PU-8580 (Silibase, Jiande, China); Castor oil (CO) (Merck Life Science, Darmstadt, Germany); Dulbecco′s Phosphate-Buffered Saline (PBS, Sigma-Aldrich, Saint Louis, MO, USA); Doxorubicin hydrochloride (DOX, Pol-Aura, Zawroty, Poland); Phosphoric acid (85%, Merck Life Science, Darmstadt, Germany), and acetonitrile (≥99.9%, Merck Life Science, Darmstadt, Germany) were all used as received. PAMAM G3 dendrimer was obtained according to Tomalia et al. [21] and purified according to Esfand and Tomalia [25], as described earlier [42].

### 2.2. Foam Preparation

The foams with 10, 20, 30, 40, or 50% PAMAM G3 mass content (P10F, P20F, P30F, P40F and P50F, respectively) were obtained according to the procedure described earlier [31,35]. Briefly, appropriate amounts of PAMAM G3 dendrimer, glycerin-based polyether polyol (with hydroxyl number value within the range of 330–360 mg KOH/g and dynamic viscosity 250–310 mPas at temp. 25 °C) and castor oil were weighed into a 200 cm^3^ polypropylene cup in the amounts given in Table 1.

The synthesis followed a previously described two-step mixing protocol [31,35], involving controlled heating (≈50 °C) to achieve a homogeneous prepolymer mixture before foaming. Then, demineralized water and silicone were added and mixed at 50 °C until a homogeneous mixture was obtained. After cooling the contents to room temperature, pMDI (Equation (1)) was added, and finally, catalyst TEA. All ingredients were vigorously mixed together until creaming occurred. After the foam had finished growing, it was left for 24 h at room temperature to dry completely. After seasoning for a week, it was used for further tests.

The reference foam (RF) was obtained according to the standard procedure.

(1) $$m_{\mathrm{NCO}}=42\;\frac{m_{p}}{p_{\mathrm{NCO}}}\left(f\;\frac{100}{M}+\frac{p_{H_{2}O}}{18}\right)k$$

where:

*m*_p_ is mass of polyol/castor oil/dendrimer agent used for foaming [g], *f*—functionality of the polyol/castor oil/dendrimer agent, *p*_NCO_—content of NCO groups in pMDI [wt%], $p_{H_{2}O}$—H_2_O content in relation to the weight of the polyol used agent [wt%], *M*—molar mass of the polyol used agent, 42—molar mass of the NCO group, 18—molar mass of water.

### 2.3. Microscopy

Microscopic images of the foams were collected with a Pantera series transmitted light microscope (Motic, Hong Kong, China) equipped with a 10× objective lens. Images were captured with the integrated digital camera in TIFF format. Representative regions were selected to illustrate the morphology, pore structure, and cell wall thickness of the foams.

### 2.4. Density and Porosity Measurements

The apparent density (*ρ*_bulk_) of studied foams was determined for cubic samples according to UNE EN ISO 845:2010 as the ratio of mass (*m*) to geometric volume (*V*) [43]. Cubic foam samples (five for each foam) were cut with a sharp blade and measured with a digital caliper (±0.01 mm) at three positions along each edge. The samples were dried to constant mass (room temperature, vacuum, 24 h) and weighed to an accuracy of ±0.1 mg.

The total porosity (ε) of the polymer–dendrimer composites was estimated from the relationship between apparent density and true density (*ρ*_solid_) (Equation (2)). A constant true density of 1.20 g/cm^3^, corresponding to the density of the solid polyurethane foam, was assumed for all formulations [44]. The bulk densities of the PU–PAMAM foams ranged from 33 to 55 kg/m^3^ (0.033–0.055 g/cm^3^), resulting in values more than twenty times lower than the assumed solid density. Consequently, moderate variations in *ρsolid* would result in changes in the calculated porosity of approximately 1%, which is within the experimental uncertainty of bulk density measurements. Therefore, the use of a constant solid density does not affect the comparative analysis of porosity between formulations.

(2) $$\varepsilon =\left(1-\frac{\rho_{\mathrm{bulk}}}{\rho_{\mathrm{solid}}}\right)\;\cdot \;100\%$$

### 2.5. Rheological Measurements

Polyurethane foams were examined in rheological creep–recovery tests. Cylindrical specimens were cut using a 20 mm steel punch, with sample heights in the range of 3–5 mm. Measurements were carried out on a HAAKE Rheostress 6000 rheometer (Thermo Scientific, Waltham, MA, USA) equipped with a plate–plate geometry (20 mm diameter). Both plates were serrated to minimize slippage. Prior to each measurement, the gap was calibrated by bringing the plates into contact under a normal force of 5 N, which ensured proper fixation of the specimen and prevented lateral displacement. The test protocol consisted of a creep phase at a constant shear stress of 1 kPa for 100 s, followed by a recovery phase of 100 s at zero stress. The creep response was fitted with the Zener (standard linear solid) model (Equation (3)), and the recovery data were described by the Weibull function (Equation (4)). Curve fitting was performed using OriginPro 2024 (OriginLab, Northampton, MA, USA) with the iterative Levenberg–Marquardt algorithm.

(3) $$J\left(t\right)=\frac{1}{E_{1}}+\frac{1}{E_{2}}\left(1-e^{-t/\tau }\right)$$

where *E*_1_ represents the instantaneous elastic modulus, *E*_2_ the delayed elastic (relaxational) modulus, and *τ* = *η*/*E*_2_ is the characteristic relaxation time.

(4) $$J\left(t\right)=J_{v}\;\cdot \;e^{-{\left(\frac{t-t_{0}}{\lambda }\right)}^{\beta }}+J_{p}$$

*J*_v_ denotes the reversible (viscoelastic) compliance, *J*_p_ the permanent deformation, *λ* the characteristic recovery time, and *β* the shape parameter of the relaxation curve.

### 2.6. Sterilization Process for PU–PAMAM Matrices and RF Foams

The irradiation process for foam samples intended for cytotoxicity studies was carried out using 9 MeV accelerated electrons at the Institute of Nuclear Chemistry and Technology (INCT) employing the “Elektronika 10/10” linear accelerator. Dosimetry was performed using a graphite calorimeter in accordance with ASTM 51631-20e1 [45].

All samples were irradiated with an absorbed dose of 25 kGy, which is the standard sterilization dose used for polymer-based biomedical materials and pharmaceutical products, in accordance with regulatory guidelines [46]. References foams (RFs) were irradiated at room temperature in an air atmosphere. DOX-loaded PU–PAMAM matrices were irradiated on dry ice to minimize potential radiation-induced degradation of the drug. According to the literature reports, medical-grade polyurethane foams exhibit limited structural changes at standard sterilization doses, while low-temperature irradiation is commonly used to preserve the stability of radiation-sensitive drugs [47,48]. Therefore, no significant alteration of the PU–PAMAM matrix or doxorubicin stability was expected under the applied sterilization conditions.

### 2.7. NMR Spectroscopy

^1^H-NMR spectra were recorded on a Bruker Avance II 500 MHz FT-NMR spectrometer (Bruker Corporation, Billerica, MA, USA) equipped with an ULTRASHIELD 500 PLUS superconducting magnet (11.74 T; Bruker BioSpin GmbH, Rheinstetten, Germany). Measurements were performed at 298 K in DMSO-d_6_. Data processing was carried out using Bruker TopSpin software (Version 4.1.0).

### 2.8. Immobilization and Release of Doxorubicin from Matrices

#### 2.8.1. DOX Encapsulation

From the P10F, P20F, P30F, P40F, P50F and RF foams, 3 samples of dimensions approximately 1 × 1 × 0.5 cm were cut out, precisely trimmed and weighed (final mass 1.0–1.5 g). Each shape was placed separately in a 5 cm^3^ Eppendorf tube, flooded with 1.5 cm^3^ of 1 mM DOX solution in ethanol and incubated for 24 h at room temperature in the absence of light. After this time, the foams were rinsed with demineralized water to remove unbound doxorubicin from the surface and dried under reduced pressure (room temperature, 3 h). Solutions after encapsulation were retained for concentration determination by HPLC.

#### 2.8.2. DOX Release from Polymer Matrices

Falcon tubes containing 30 cm^3^ of PBS buffer pH 7.4 were placed in an orbital shaking incubator (Incu-Shaker Mini, Benchmark Scientific, Sayreville, NJ, USA) and heated to 37 °C. Then, one polyurethane foam sample was placed in each tube. Release studies were carried out for 90 h. The 90 h observation window was chosen to mimic the clinically relevant early period of use of local delivery systems such as implants, patches, or bioactive wound dressings, during which the local drug concentration is expected to be most critical [49,50]. Samples of the solutions (500 µL) containing released DOX at appropriate time intervals were collected, and an equivalent volume of PBS was added. The collected samples were analyzed for DOX content. Release experiments were conducted under sink conditions by using a sufficiently large volume of release medium and regularly replacing it. Under these conditions, the concentration of doxorubicin in the release medium remained well below its saturation solubility, and the release process was not limited by the drug’s solubility.

#### 2.8.3. Quantitative Studies of DOX Encapsulation and Release Using HPLC with UV–Vis Detection

Quantitative studies of the DOX encapsulation and release process using the HPLC method were carried out based on the procedure described by Hajare et al. [51]. A 1000 μM DOX stock solution was prepared in the eluent solution. DOX standard solutions of 20, 40, 60, 80 and 100 μM were prepared by appropriate dilution of the stock solution in the eluent solution. DOX solutions from encapsulation and release were analyzed on an HPLC chromatograph (Agilent 1100 Series, Agilent Technologies, Waldbronn, Germany) with a variable wavelength UV–Vis detector (VWD), using a standard C18 reverse phase column (Supelco Inc., Kromasil, C18 5 μm, 250 mm × 4.6 mm; Eka Chemicals AB, Bohus, Sweden). A mixture of acetonitrile and 0.01 M solution of orthophosphoric acid in water in a volume ratio of 4:6 (pH = 2) was used as the mobile phase. DOX solution samples were brought to room temperature and passed through the column in an amount of 20 µL at a flow rate of 1 cm^3^/min. Chromatograms were recorded at a wavelength of 234 nm, and the surface area was determined for the DOX signal with a retention time of 2.7 min. The values of two coefficients were determined: loading capacity and DOX encapsulation efficiency. In the literature, drug loading capacity (LC%) for dendrimers is typically expressed relative to the mass of the native carrier [52]. However, in our case, PAMAM G3 was covalently bound in the resulting polyurethane-dendrimer composite, meaning the active carrier constituted 10, 20, 30, 40 or 50% of the scaffold. Based on the difference in concentrations between the encapsulation solution and the solutions after encapsulation, the mass of bound drug was calculated for RF and PU–PAMAM foams, and then the DOX loading capacity and encapsulation efficiency were determined using Formulas (5) and (6).

(5) $$\mathrm{LC}=\frac{\mathrm{weight}\mathrm{of}\mathrm{loaded}\mathrm{drug}\left[\text{µ}g\right]}{\mathrm{weight}\mathrm{of}\mathrm{foam}\mathrm{sample}\left[\text{µ}g\right]}\cdot 100\%$$

(6) $$\mathrm{EE}=\frac{\mathrm{weight}\mathrm{of}\mathrm{loaded}\mathrm{drug}\left[\text{µ}g\right]}{\mathrm{weight}\mathrm{of}\mathrm{initially}\mathrm{used}\mathrm{drug}\left[\text{µ}g\right]}\cdot 100\%$$

The cumulative release of doxorubicin was calculated by summing the amount of drug released at each sampling time and expressing it as a percentage of the initially encapsulated drug. Calculations were corrected for the sampling volume and medium replacement at each time point. The total amount of encapsulated doxorubicin was determined experimentally based on the difference between the initial DOX concentration in the loading solution and the concentration measured after the encapsulation process.

### 2.9. Cell Culture

Human immortalized keratinocytes (HaCaT) provided by CLS Cell Lines Service GmbH (Eppelheim, Germany) were cultured in DMEM medium, and human squamous carcinoma cells (SCC-15) purchased in American Type Culture Collection (Manassas, VA, USA) were grown in DMEM/F-12 supplemented with hydrocortisone (400 ng/mL). Both culture media were supplemented with 10% inactivated FBS and 100 U/mL penicillin 100 µg/mL streptomycin solution. Cells were incubated at 37 °C in humidified 95% air, 5% CO_2_, with media changed every 2–3 days. Cells were passaged at 70–85% confluence after trypsinization with 0.25% trypsin-EDTA in PBS (without calcium and magnesium ions). Cell morphology was evaluated with a Nikon TE2000S Inverted Microscope with phase contrast (Tokyo, Japan). Number and viability of cells were estimated by trypan blue exclusion test using an Automatic Cell Counter TC20 (BioRad Laboratories, Hercules, CA, USA) or Neubauer chamber.

### 2.10. Extract and DOX Cytotoxicity Assay with Neutral Red (NR)

The extracts of studied foams were prepared in accordance with ISO 10993-12 guidelines [53] in complete culture medium DMEM/F12. Four sterile foam pieces (1 × 1 × 0.5 cm^3^) were totally immersed in 5.3 mL complete DMEM/F12 medium and incubated at 37 °C for 72 h on a shaker. Sterile extracts were diluted in complete medium to 3–100% concentrations. HaCaT and SCC-15 cells were seeded into 96-well plates (5 × 10^3^ cells/well) and incubated for 24 h at 37 °C. Then, medium was replaced with obtained extracts (100 μL/well) or DOX solutions, and cells were incubated for 72 h (37 °C, 5% CO_2_). As a control, cells without extracts were used. Then, medium was replaced by 2% neutral red in the full culture medium (100 μL/well), and cells were incubated for 1 h. After rinsing with warm PBS, 100 μL/well of fixative (50% ethanol, 49% H_2_O and 1% glacial acetic acid) was added, and plates were shaken until complete dye dissolution (450 rpm, 15 min, room temperature). Absorbance was measured at 540 nm and 620 nm with a microtiter plate reader (μQuant–BioTek, Winooski, VT, USA) against a blank sample (fixative without cells). Percentage cell viability was calculated by normalization of the absorbance readings against that of the non-treated cells (set as 100%). Three independent experiments were performed in triplicate.

### 2.11. Statistical Analysis

Due to the lack of normal distribution in the experimental groups, statistical analysis was performed using the non-parametric Kruskal–Wallis test to estimate the differences between the extract-treated cells and non-treated control in each cell line. *p* ≤ 0.05 was considered as statistically significant. Calculations were performed using Statistica 13 (StatSoft).

## 3. Results and Discussion

### 3.1. Preparation of PU–PAMAM Matrices Containing 10, 20, 30, 40, or 50 Mass % of PAMAM G3 Dendrimer Nanoparticles

As mentioned earlier, the first successful attempt to obtain foam with PAMAM G3 dendrimer nanoparticles was described in our previous paper [41]. Due to the target use of the material as a matrix for encapsulation and release of drugs, the research consisted in developing a composition with the highest possible amount of PAMAM G3 dendrimer. The result of the research was the development of a methodology for obtaining PU–PAMAM foam containing 50 wt% PAMAM G3 and optimization of the mixture composition. Each of the components used for the synthesis of this foam had its own individual task. The dendrimer acted as an active ingredient capable of encapsulating drugs. Rokopol G441 was a polyol component that liquefied the dendrimer and allowed its subsequent homogenization with the remaining components of the composition. This was a key component, without which obtaining foam with a dendrimer was impossible. Castor oil (CO) in a mass concentration of 15% improved the homogenization and morphology of the obtained foams. Water acted as a foaming agent, while triethylamine was the reaction catalyst. However, DOX release and uptake, and high cytotoxicity of PU-matrices containing 50 wt% PAMAM G3, showed that the amount of dendrimer should be reduced.

Therefore, using the same procedure, foams with 10–50 wt% PAMAM G3 dendrimer were prepared. PU–PAMAM foams with 10–50% by mass of PAMAM G3 dendrimer (P10F, P20F, P30F, P40F, or P50F, respectively) were obtained on a 4 g scale of multifunctional substrates: G441, CO and PAMAM G3. For comparative purposes, a reference foam (RF) was also prepared. For foams containing 10–40% by weight of PAMAM G3, the effect of castor oil on the morphology was the same as in the case of P50F foam. Therefore, 15% by weight was also used. The required amount of water and surfactant that resulted in optimal foam growth also remained at the same level as for the P50F foam. However, as the PAMAM G3 content in the mixture decreased, the amount of catalyst had to be reduced. For P50F and P40F, the amounts were the same, for P30F they were reduced to two-thirds of the initial value, and for lower PAMAM and RF concentrations, they were reduced by half.

The reference foam was characterized by regular arrangement of pores and the shortest cream and growth times (Table 1). On the other hand, foams containing dendrimer in their composition had irregular pores and were more compact. Their growth and cream times rose with the increase in dendrimer content in the composition.

### 3.2. Structure of Obtained Matrices

**Figure 1:**
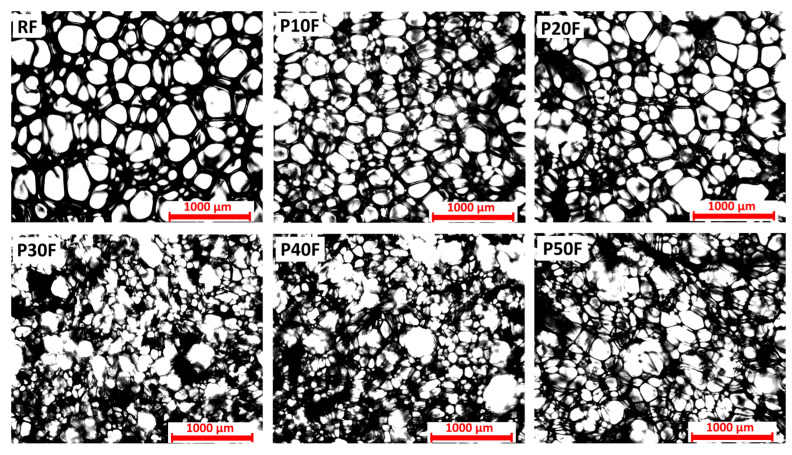
Porous structure of polyurethane foams (RF) and polyurethane–dendrimer composite containing 10% (P10F), 20% (P20F), 30% (P30F), 40% (P40F), or 50% (P50F) by mass of PAMAM G3 dendrimer. Images obtained with transmitted light microscope. Scale equals 1000 µm.

Microscopic images of PU–PAMAM foams containing 10, 20, 30, 40, or 50 mass % of PAMAM G3 dendrimer nanoparticles revealed significant differences in the porous structure resulting from different amounts of dendrimer in the material composition (Figure 1).

The dendrimer-free reference foam, containing only polyether polyol as well as castor oil as co-components, was characterized by a relatively uniform and compact structure. The addition of PAMAM G3 affected the structure depending on the dendrimer content. Up to 20 wt% of dendrimer, the cells were well-developed (apart from smaller sizes and few visible clusters), and their structure was similar to that of polyurethane foam without PAMAM G3 dendrimer. This morphology suggests the foaming process proceeded correctly, without interference from the presence of dendrimer nanoparticles in the system. However, from a concentration of 30 wt% PAMAM G3, the structure of PU–PAMAM composites differed significantly from RF foam and foams with 10% and 20% PAMAM G3 concentrations. In samples P30F and P40F, the cells were irregular and characterized by a wide size distribution. Broken and disconnected cells were visible. The porosity became more pronounced, with evident thinning of the cell walls and an increase in the number of broken or partially fused pores, indicating a weakening of the structural integrity. The highest dendrimer concentration (P50F) led to highly irregular, open-cell structures with large interconnected pores and a significantly disrupted structure. This suggests that excessive PAMAM content negatively affects foam stability, likely due to interference with the polymer cross-linking or foaming process. The observed differences confirm that the PAMAM G3 content is a key parameter shaping the internal structure of the polymer matrix. Therefore, considering the structure, PAMAM G3 dendrimer up to 20 wt% allows for a well-developed porous structure without pore defects. The observed differences confirm that the share of PAMAM G3 is a key parameter shaping the internal structure of the polymer matrix.

### 3.3. Porosity of Foams

The very high porosity values (approximately 97%) of the PU–PAMAM G3 matrices indicate that only ~3% of the total foam volume is a solid polymer–dendrimer network, with the remainder being interconnected pores (Table 2). Such high porosity is characteristic of open-cell foams intended for biomedical applications, where values of 95–98% are often targeted to promote cell infiltration and efficient mass transport [54,55]. The slight difference in apparent density between PU–PAMAM foams is due to changes in foam cell size and wall thickness resulting from the higher PAMAM content. Therefore, the porosity values determined for these PAMAM-modified polyurethane foams are in the expected range for high-performance open-cell polymer scaffolds. In both cases, high porosity is beneficial for drug delivery purposes because it provides a large internal surface area and facilitates rapid penetration of the release medium, whereas small differences in density can affect mechanical stability and drug loading uniformity.

### 3.4. Creep–Recovery Behavior of PU–PAMAM Foams

**Figure 2:**
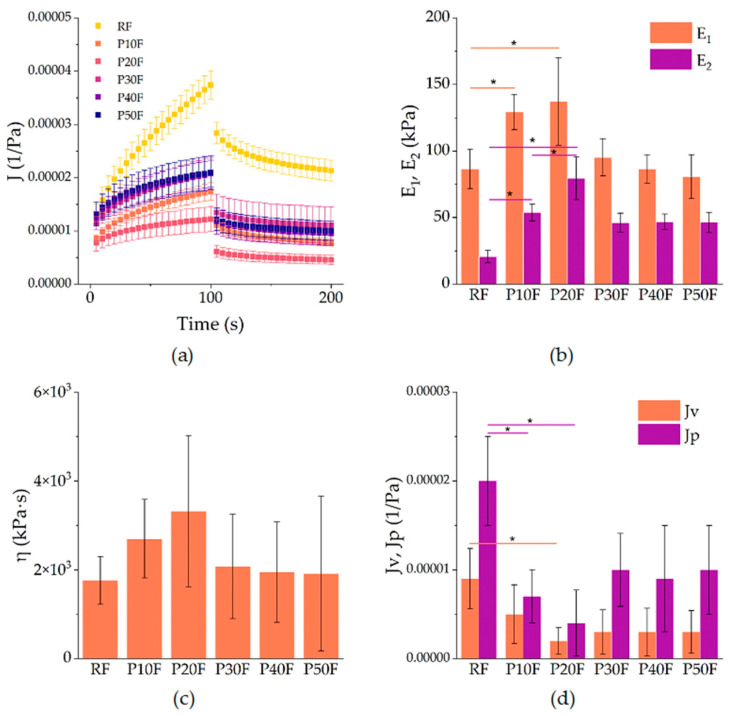
(**a**) Creep–recovery compliance *J (t)* of neat polyurethane foam (RF) and P10F–P50F modified foams at 1 kPa shear stress. Symbols represent mean values, error bars denote standard deviations. (**b**) Parameters obtained from fitting: elastic moduli *E*_1_ and *E*_2_ from the Zener model, (**c**) viscosity *η* of the Maxwell branch, and (**d**) recoverable (*J*_v_) and non-recoverable (*J*_p_) compliance from the Weibull function. Asterisks indicate statistically significant differences between groups (*p* < 0.05).

The creep–recovery curves obtained for the reference polyurethane foam (RF) and the PAMAM G3-modified foams (P10F–P50F) revealed a typical viscoelastic response characteristic of polymeric foams (Figure 2a).

During the creep stage, all samples showed an increase in compliance with time, followed by a partial recovery after removal of the load. The incorporation of PAMAM G3 dendrimer markedly altered the balance between elastic and viscous contributions. The RF foam exhibited the highest compliance values, indicating lower stiffness and more pronounced deformation under load. In contrast, foams with 10–20% PAMAM G3 (P10F and P20F) showed reduced compliance, indicating enhanced resistance to creep and improved elastic recovery. At higher dendrimer contents (P30F–P50F), the curves again shifted towards higher compliance, suggesting a loss of reinforcement efficiency and a return to more compliant, softer behavior.

The creep data were described by the Zener model, which provides two elastic moduli: the instantaneous modulus *E*_1_ and the long-term modulus *E*_2_, as well as the viscosity *η* of the Maxwell branch. The Zener model revealed that PAMAM G3 increased the elastic response up to 20% loading (Figure 2b,c). Specifically, E_1_ rose from approximately 86 kPa in RF to 129 kPa in P10F and 137 kPa in P20F, corresponding to an improvement of ca. 50–60%. A similar trend was observed for E_2_, which increased from 21 kPa in RF to 54 kPa in P10F (+160%) and 80 kPa in P20F (+285%). Concomitantly, the viscosity *η* also rose, from 1.76 × 10^6^ Pa·s for RF to 2.70 × 10^6^ Pa·s in P10F and 3.32 × 10^6^ Pa·s in P20F, highlighting a strong suppression of time-dependent deformation. These findings confirm that moderate PAMAM G3 incorporation reinforced the foams mechanically. However, higher loadings (≥30%) led to a drop in both *E*_1_ and *E*_2_, with values returning close to those of RF (e.g., *E*_1_ ~95 kPa and *E*_2_ ~46 kPa for P30F, and lower for P40F and P50F), accompanied by a decrease in viscosity to ~1.9–2.1 × 10^6^ Pa·s. Thus, mechanical improvement was limited to ≤20% dendrimer content, whereas higher loadings led to property deterioration.

The recovery phase was successfully fitted with the Weibull function, yielding parameters that describe the recoverable compliance *J*_v_, the non-recoverable compliance *J*_p_, and the kinetics of recovery (*λ*, *β*). As shown in Figure 2d, the RF foam exhibited the largest values of both *J*_v_ (9 × 10^−6^ 1/Pa) and J_p_ (2 × 10^−5^ 1/Pa), indicating limited elastic recovery and pronounced irreversible deformation. Modification with 10% PAMAM G3 reduced these parameters substantially: *J*_v_ decreased to 5 × 10^−6^ (−45% vs. RF) and *J*_p_ to 7 × 10^−6^ (−65% vs. RF). The effect was even stronger in P20F, with *J*_v_ = 2 × 10^−6^ and *J*_p_ = 4 × 10^−6^, corresponding to ca. 75–80% reduction relative to the reference. At higher dendrimer contents (P30F–P50F), both *J*_v_ and *J*_p_ increased again to levels comparable to RF, which corroborates the decrease in elastic reinforcement observed in creep. The Weibull parameters *λ* (50–66 s) and *β* (0.69–0.76) remained within narrow ranges and did not show statistically relevant differences, so they were not further discussed.

### 3.5. Calculation of DOX Loading Capacity and Encapsulation Efficiency

The encapsulation and release methodology was developed based on available literature [56,57,58]. Given the susceptibility of DOX to self-aggregation and dimer formation in aqueous and buffered environments such as PBS [59], ethanol was used as a solvent of choice for DOX during encapsulation. As a result of the chromatographic measurements of DOX standard solution samples, the surface area for each standard solution was determined. The calibration curve showed straightness in the tested concentration range (0–100 µM), with a slope of 49.32 and a determination coefficient of R^2^ = 0.9996.

The results regarding loading capacity and encapsulation efficiency showed a significant effect of the presence of PAMAM G3 dendrimer on the ability of PU–PAMAM foams to bind DOX. The reference RF sample without dendrimer showed lower values of both LC (7.7%) and EE (11%) compared to PU–PAMAM G3 foams, with LC values ranging from 11 to 35.3% and EE from 14 to 51% (Table 3).

Both parameters increased with increasing PAMAM G3 content up to 40 wt%, reaching a maximum in sample P40F, and then decreased in the sample with the highest dendrimer content. This trend can be attributed to the increasing amount of PAMAM G3 nanocarrier as well as the formation of an open, highly interconnected porous structure that facilitates the diffusion and adsorption of DOX at moderate PAMAM G3 loading. At higher dendrimer loading, partial pore coalescence and increased structural density likely limit drug penetration and binding, resulting in significantly lower LC and EE values.

The obtained LC values for PU–PAMAM G3 were within the range reported for native PAMAM G3–G4 dendrimers loaded with doxorubicin (DOX) (typically 5–40%) [60,61], despite the fact that in our case, the dendrimers were covalently cross-linked within the polyurethane network. This suggests that although cross-linking partially restricts access to the interior of the cavity, the porous structure of the matrices enables efficient DOX encapsulation. The obtained results indicate that a moderate PAMAM G3 loading maximizes the drug loading benefits, outperforming both unmodified matrix systems and native dendrimers. This confirms the suitability of PAMAM G3 dendrimers as effective passive encapsulation enhancers in polymeric drug delivery matrices.

### 3.6. Correlation Between Structure, Rheological Behavior and Drug Loading Efficiency of PU–PAMAM Foams

**Figure 3:**
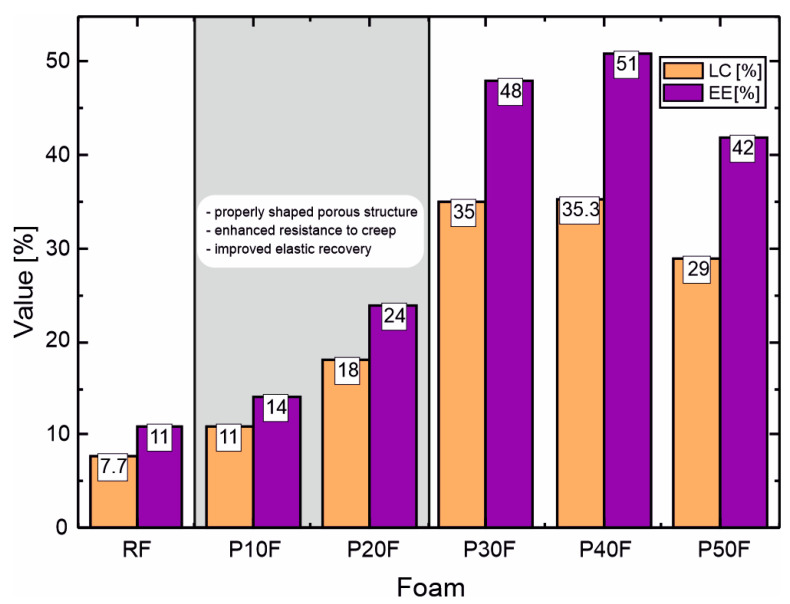
Comparison of loading capacity (LC) and encapsulation efficiency (EE) of PU–PAMAM and RF foams with indication of the optimal range of PAMAM G3 content, taking into account the structure and rheological properties of the matrices.

The structural, rheological, and DOX-encapsulation parameters of studied foams clearly indicate that the PAMAM G3 present in matrices plays a key role in determining the structure–property relationships of the resulting matrices. Microscopic observations showed that up to 20 wt% PAMAM G3 foams retained a homogeneous open-cell structure with well-developed and interconnected pores, similarly to the reference polyurethane foam, but with smaller and more uniform cells. At higher dendrimer concentrations (≥30 wt%), partial pore coalescence, cell wall thinning, and the formation of irregular and partially collapsed pores were observed, suggesting that excessive dendrimer loading interferes with the foaming and cross-linking processes. Rheological studies confirmed these morphological results. Adding PAMAM G3 at 20 wt% of matrices resulted in a significant reduction in the overall foaming and cross-linking properties, an increase in elastic modulus, and a reduction in creep compliance, indicating improved structural integrity and stronger internal interactions between polymer chains. Above this concentration, viscous deformation dominated the viscoelastic response, reflecting weakening of the polymer network. The encapsulation results agree well with the structural and mechanical observations. Although formulations containing 30–40 wt% PAMAM G3 were characterized by the highest LC and EE values, they showed a clear deterioration of the porous structure and reduced viscoelastic stability compared to P10F and P20F matrices. Moreover, as shown in the following sections, dendrimer contents in PU–PAMAM matrices in the range of 30–50 wt% are associated with reduced matrix integrity in vitro, a burst release profile, and excessive cytotoxicity. Therefore, considering the combined structural, mechanical, and functional parameters, matrices containing 10–20 wt% PAMAM G3 represent the most balanced composition (Figure 3).

### 3.7. DOX Release Profiles

**Figure 4:**
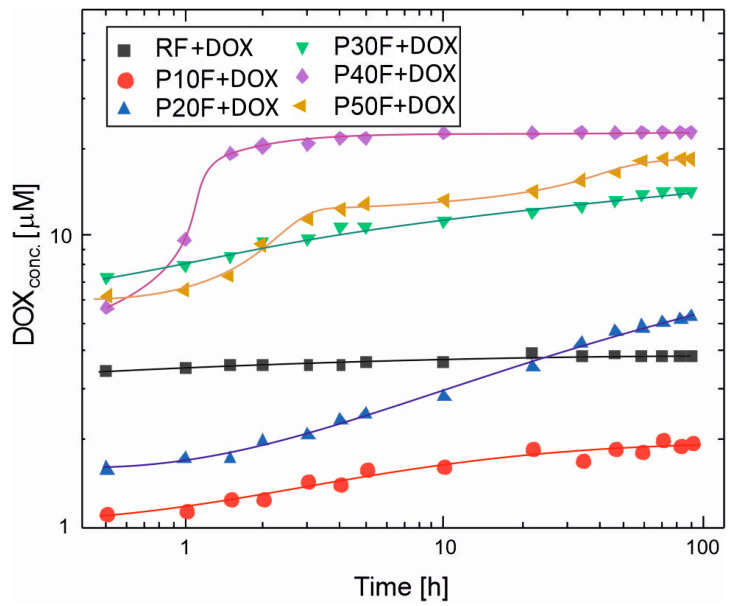
The DOX release profiles from PU–PAMAM G3 foams and the reference foam (RF) in phosphate-buffered saline (PBS, pH 7.4) at 37 °C after 90 h. Each point is an average from three independent experiments. Whiskers show standard deviations. The %RSD (Relative Standard Deviation) for measures was under 3%, which complies with the Pharmacopoeia standards for pharmaceutical applications [62].

Based on HPLC analysis of the DOX solution from the process of its release from the reference foam and foams with PAMAM G3 dendrimer, DOX concentrations in each sample were calculated. This allowed for the preparation of graphs of the dependence of DOX concentration in the release medium on time and the determination of release profiles (Figure 4). For all foams, the most pronounced changes in DOX concentration occurred within the first 48–72 h, followed by minor variations at longer times. Therefore, the selected 90 h observation period was sufficient to capture both the initial burst and the early sustained release phases relevant for local drug delivery applications.

The release profiles of doxorubicin from the polyurethane–dendrimer foams revealed that the PAMAM G3 presence had a crucial influence on the drug diffusion rate. Dendrimer-free RF foam released DOX with over 3.5 μM concentration in PBS after 0.5 h, and the DOX concentration did not change significantly until the end of observation. This suggests that equilibrium between drug absorption and desorption between the foam and the release medium was reached very quickly. The reference foam exhibited a burst release profile, typical of porous matrices without specific drug-carrier interactions. The rapid achievement of equilibrium concentration suggests that DOX molecules were primarily adsorbed to the surface and poorly controlled within the polymer network. This behavior has been described for hydrogels and porous polymer systems lacking functional binding sites [63,64,65].

In contrast, excluding P10F, PU–PAMAM foams achieved higher peak concentrations, which resulted from the encapsulation of DOX within the PAMAM G3 structure. PAMAM G3–DOX interactions retarded diffusion, resulting in gradual desorption of the drug. In general, for PU–PAMAM foams, the range of initial and final concentrations in the medium correlates with the LC and EE values, and the release profiles with the structural-rheological properties (Figure 3). The P40F matrix released the highest DOX concentration in PBS among all PU–PAMAM foams and also demonstrated burst DOX release, reaching 19 μM after 1.5 h and then stabilizing at ~22 μM after 4 h.

The P30F and P50F matrices released DOX to a slightly lower degree, resulting in concentrations in the range of 7.3–14 μM for P30F and 6.2–18.4 μM for P50F in PBS after 0.5 h. A significantly smaller difference in initial and final concentrations compared to the P40F matrix (5.6–22 μM) was observed. The release results achieved for the matrices with dendrimer loadings ≥30 wt% were a consequence of pore coalescence, which likely led to the formation of preferential diffusion paths and reduced diffusion resistance. The decrease in mechanical integrity observed in rheological tests for these compositions further supports the hypothesis that excess PAMAM disrupts the cross-linked network and alters solvent permeability.

The P10F and P20F foams exhibited the most uniform and predictable release patterns, consistent with their regular pore architecture and balanced viscoelasticity. Figure 4 shows that for P10F and P20F foams, there is an initial release of DOX with the dynamic concentration increase for P20F taking longer than for P10. The DOX released from P10F reached a concentration above 1 μM after 0.5 h with equilibrium at ~1.8 μM after about 22 h. The DOX concentration released from P20F reached about 1.5 μM after 0.5 h incubation, which continued to increase up to 58 h, reaching a value of 4.8 μM, and increased up to 90 h.

**Figure 5:**
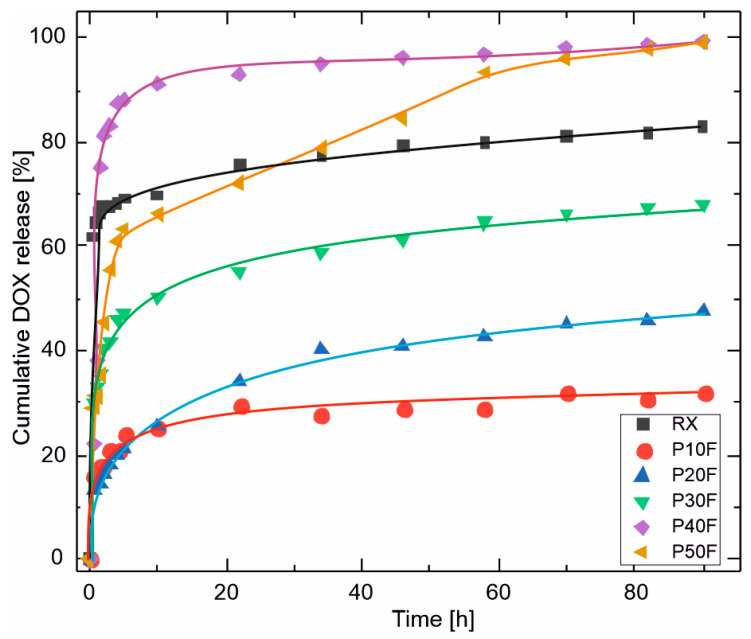
Cumulative release of doxorubicin (DOX) from PU–PAMAM foams with different PAMAM G3 contents, expressed as a percentage of the initially encapsulated drug, as a function of time. The profiles highlight pronounced differences in both release kinetics and final release extent, ranging from diffusion-limited partial release for low-PAMAM foams (P10F–P20F) to burst-dominated, near-complete release for high-PAMAM formulations (P40F–P50F).

To enable direct comparison of the release behavior between PU–PAMAM matrices with different PAMAM G3 content, doxorubicin release was additionally analyzed in terms of cumulative release expressed as a percentage of the initially encapsulated drug (Figure 5).

The cumulative doxorubicin release obtained for the PU–PAMAM foams spanned a broad range, from approximately 32% to nearly 100% after 90 h, depending on the PAMAM G3 content. Low-PAMAM foams (P10F and P20F) released only 30–47% of the loaded drug, indicating partial retention of DOX within the PU–PAMAM network. Intermediate PAMAM content (P30F) resulted in approximately 68% cumulative release, while high PAMAM contents (P40F and P50F) led to nearly complete drug release. The reference foam reached a plateau at approximately 83% release. Such variability is well aligned with literature reports on polymer-based local drug delivery systems, where the final fraction of released drug is strongly governed by matrix porosity, drug–polymer interactions, and the dominant release mechanism [66,67,68,69,70].

Because the release studies were performed under sink conditions, the observed differences in cumulative release reflect differences in matrix structure and drug–polymer interactions rather than limitations imposed by drug solubility. The near-complete release observed for P40F and P50F suggests that, at high PAMAM loadings, doxorubicin is predominantly weakly associated with the matrix, facilitating rapid desorption and diffusion. Conversely, lower PAMAM contents provide stronger retention and more controlled release due to a combination of restricted pore connectivity and stronger dendrimer–drug interactions.

The obtained data for the DOX release were fitted to several kinetic models (zero-order, Higuchi, Korsmeyer–Peppas [71,72]). It was found that the latter model best describes the experimental data. Values of release exponents, *n*, and coefficients of determination, R^2^, are listed in the Table 4.

The Korsmeyer–Peppas model uses a semi-empirical relationship to describe drug release from a polymeric system over time:

(7) $$\frac{m_{t}}{m_{0}}=k\cdot t^{n}$$

where *m*_0_ is the total mass of drug, *m*_t_ is the mass of drug released at time *t,* and *k* is the release rate constant.

Obtained results from curve fitting indicate a non-Fickian diffusion-controlled mechanism.

Importantly, near-complete release should not be interpreted as superior performance. Although high PAMAM contents maximize drug availability, this occurs at the expense of release control and structural stability and may be associated with a higher initial burst. In contrast, foams containing 10–20 wt% PAMAM G3 provide a more balanced release behavior, combining sufficient drug loading with controlled kinetics and favorable structural integrity. These findings demonstrate that the release of DOX from PU–PAMAM foams is governed by the interplay between matrix porosity and dendrimer–drug affinity and confirm that optimal performance corresponds to an intermediate PAMAM G3 content rather than maximal drug release.

Moreover, the incorporation of PAMAM G3 into polyurethane foams modified with castor oil (CO) and polyether polyol (G441) enhanced resistance to creep and improved elastic recovery up to 20% content. Beyond this concentration, the beneficial effect was lost, and the foams become softer and more compliant, resembling the behavior of the reference system. These results confirm that moderate incorporation of PAMAM G3 provides an optimal balance of urea and urethane cross-links, leading to improved viscoelastic performance.

**Figure 6:**
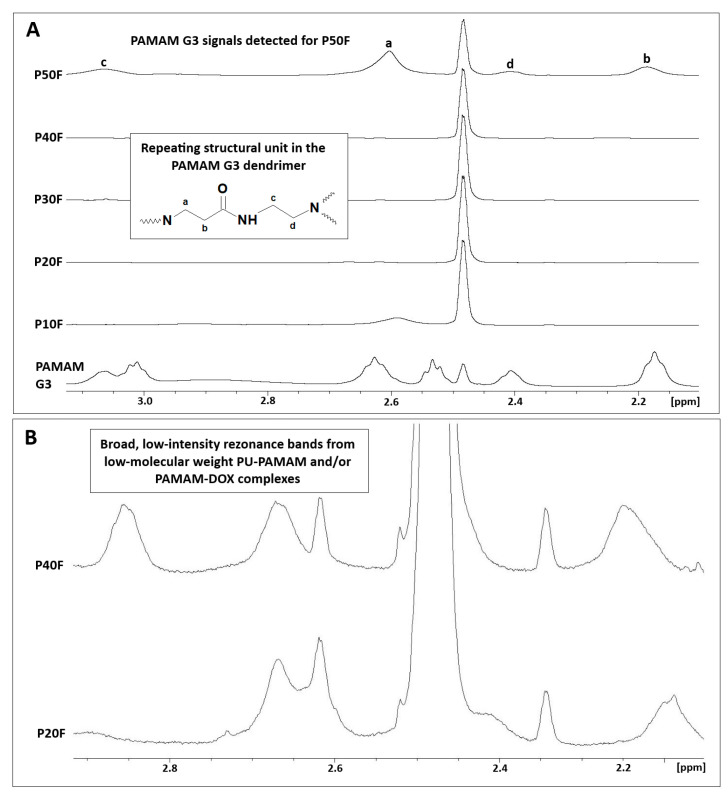
(**A**) ^1^H-NMR spectra of PBS solutions collected after DOX release from polyurethane matrices containing 10–50 wt% PAMAM G3 (DMSO-d_6_, 500 MHz) compared to native dendrimer. Signals labeled a–d correspond to characteristic methylene protons of the PAMAM G3 repeating unit, as indicated in the structural inset. (**B**) Enlarged regions of the ^1^H-NMR spectra showing broad, low-intensity resonance bands attributed to low-molecular-weight PU–PAMAM species and/or PAMAM–DOX complexes released into the PBS solution.

The ^1^H-NMR spectra of PBS solutions collected after DOX release from the P10F–P50F polyurethane matrices revealed the presence of PAMAM G3 in the P50F matrix extract (Figure 6).

Characteristic broad resonance bands corresponding to the methylene protons of PAMAM G3 (a–d), attributed to –CH_2_– groups adjacent to amide moieties and tertiary amines, were observed in the 2.1–3.1 ppm range. This confirms partial dendrimer release at the highest PAMAM G3 loading. ^1^H-NMR spectra of buffer extracts after DOX release from samples with lower dendrimer loading (P20F–P40F) showed the presence of broad, low-intensity resonance bands in the 2.0–2.9 ppm range, suggesting the presence of low-molecular-weight PU–PAMAM species and/or PAMAM–DOX complexes.

### 3.8. Cytotoxicity

Toxicity studies were conducted using the indirect neutral red (NR) assay. This assay was chosen because it assesses not only toxicity itself but also an effect on proliferation rate. Doxorubicin (DOX) is a drug whose mechanism of action is primarily based on blocking cell division [35]. However, in the direct contact assay, cells do not divide because they are cultured on 100% confluence [73].

**Figure 7:**
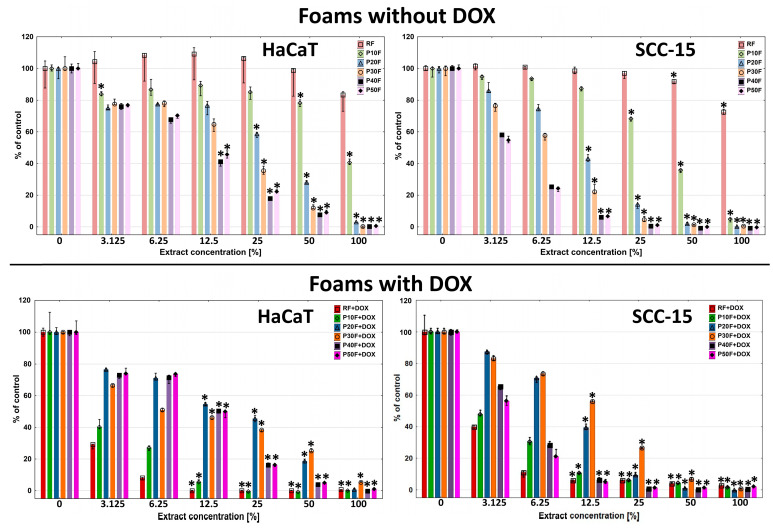
Viability of immortalized human keratinocytes (HaCaT) and human squamous cell carcinoma cells (SCC-15) after 72 h of incubation with extracts from native PU-foams and DOX containing matrices incubated with 1 mM DOX solution in ethanol. Data estimated with NR assay. Cell viability was expressed as percent of the non-treated control (medians). The whiskers are lower (25%) and upper (75%) quartile ranges. * *p* ≤ 0.05; Kruskal–Wallis test (against non-treated control).

Toxicity studies performed with the NR assay showed that the most biocompatible foam was that containing no PAMAM G3 dendrimer–RF. It showed no toxicity against the normal cell model HaCaT cells across the entire concentration range, but only against SCC-15 cells incubated with 50% and 100% concentrations of extract (Figure 7).

These results were consistent with those obtained previously [41]. The P10F foams exhibited slightly higher cytotoxicity, causing a significant decrease in HaCaT and SCC-15 cell viability from 50% and 25% concentration, respectively. Higher PAMAM G3 dendrimer content in the foams resulted in increasingly higher levels of matrix toxicity, which at 100% extract concentration, resulted in 100% cell death in both cell lines. It is also worth mentioning that the toxicity of PU–PAMAM and RF foams was always higher against cancer than against non-cancerous cells.

We made a hypothesis that the concentration-dependent cytotoxicity of the extracts (P20F–P50F) was probably associated with the leaching of cationic PU–PAMAM fragments, since NMR analysis revealed that their amount increased with the PAMAM G3 content in the foams, and in the case of the P50F matrix, additionally with the presence of free PAMAM G3. Cationic PAMAM dendrimers are considered as toxic [74]. We assumed that incorporating dendrimers into the matrix structure would solve the problem of their toxicity, but as we observed, the dendrimer was not completely incorporated into the matrix structure and after release, it and its fragments were a factor inducing cytotoxicity.

The release of small dendrimer fragments from the matrices had significance. The RF + DOX, P10F + DOX, P20F + DOX, and P30F + DOX foams with immobilized DOX were significantly more toxic to HaCaT cells. However, P40F + DOX and P50F + DOX exhibited a very similar cytotoxicity profile to the analogues without DOX. A reduction in the viability of SCC-15 cells treated with DOX-containing foam extracts was observed only with RF + DOX and P10F + DOX matrices compared to the native matrices. Only a weak DOX-induced enhancement effect was observed in the P20F matrix. Interestingly, the presence of DOX in the P30F matrices resulted in decreased toxicity against SCC-15 cells.

We hypothesized that the PAMAM G3 fragments released from the matrices containing it to the highest degree, forming complexes with DOX, reduced its effect on the studied cells. This, in turn, prevented DOX from inducing additional toxicity.

**Figure 8:**
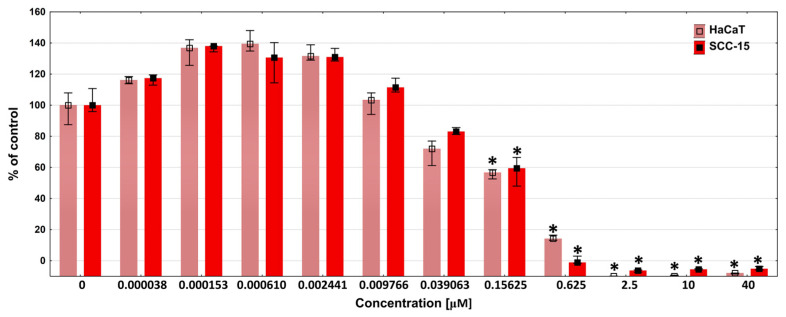
Cytotoxicity of DOX in concentrations in the range of 0–40 µM against HaCaT and SCC-15 cells after 72 h incubation, estimated with NR assay. Results are expressed as percent of the non-treated control (medians). The whiskers are lower (25%) and upper (75%) quartile ranges. * *p* ≤ 0.05; Kruskal–Wallis test (against non-treated control).

To explain this observation, we performed a DOX cytotoxicity assay against HaCaT and SCC-15 cells. The obtained results show that both cell lines responded similarly to DOX. A statistically noticeable decrease in cell viability was observed from a concentration of 39 nM, and a statistically significant decrease from 156 nM. Cell viability dropped to zero at a 2.5 µM DOX concentration (Figure 8).

The above-mentioned results of the DOX release studies from dendritic matrices showed that the DOX concentration after 72 h in PBS solution ranged from approximately 2 µM for the P10F + DOX foam to 20 µM for the P40F + DOX foam. Considering the obtained results, all foams should have caused a decrease in the viability of the tested cells to practically zero in a 100% extract. In the case of the P40F + DOX matrix, even a 3% extract should have killed almost all cells. However, this effect was not observed in either cancer or immortalized cells, where viability was above 60%. This suggests that DOX may form complexes with dendrimer fragments released from the matrix and exert significantly weaker activity. This effect was previously observed by Szota et al. [75].

Furthermore, our studies showed that DOX at low, nanomolar concentrations (0.15–2.4 nM) induced an increase in cell viability. Therefore, if we assume that some of the DOX was bound by dendrimer fragments released from the matrices and, consequently, the concentration of free DOX in solution decreased, the decrease in cell viability could have been significantly weaker than expected. This also confirms our previous hypothesis. However, this phenomenon needs deeper study.

In summary, RF, P10F and partially P20F dendrimer PAMAM G3-containing matrices seem to be useful tools for the release of the anticancer drug DOX, due to their low toxicity. Moreover, they were slightly more toxic against cancer cells, and the DOX released from the matrices exerted an anticancer effect.

## 4. Conclusions

This study shows that PAMAM G3 content strongly influences the structure, mechanical behavior, and doxorubicin loading and release of polyurethane foams. While higher dendrimer contents increase drug loading and release, excessive PAMAM leads to structural heterogeneity, reduced mechanical stability, and increased cytotoxicity. A multiparameter analysis identified 10–20 wt% PAMAM G3 as the optimal range, providing the best balance between structural integrity, controlled release, and biocompatibility. These findings demonstrate that optimal performance does not coincide with maximal drug loading or release, but rather with a balanced intermediate composition.

Therefore polyurethane–dendrimer hybrid matrices with the right proportions of contents represent a promising platform for the development of controlled local drug delivery systems, particularly for anticancer or wound healing applications.

## Figures and Tables

**Table 1:** Quantities of individual components used for the synthesis of PU–PAMAM foams with different weight fractions of PAMAM G3 dendrimer and reference foam (RF) as well as the foaming process.

Substrate Ratio [wt%]	Sample	Substrates							Foaming Process	
		G3 [g]	G441 [g]	CO [g]	pMDI [g]	H_2_O [g]	Silicone [g]	TEA [g]	Cream Time [s]	Time of Expanding [s]
G3:G441:CO 50%:35%:15%	P50F	2.0	1.4	0.6	3.57	0.16	0.16	0.068	15	125
G3:G441:CO 40%:45%:15%	P40F	1.6	1.8	0.6	3.64	0.16	0.16	0.068	14	120
G3:G441:CO 30%:55%:15%	P30F	1.2	2.2	0.6	3.71	0.16	0.16	0.051	13	100
G3:G441:CO 20%:65%:15%	P20F	0.8	2.6	0.6	3.77	0.16	0.16	0.034	12	50
G3:G441:CO 10%:75%:15%	P10F	0.4	3.0	0.6	3.84	0.16	0.16	0.034	11	47
G441:CO 85%:15%	RF	-	3.4	0.6	3.91	0.16	0.16	0.034	10	40

**Table 2:** Apparent density and porosity calculations in P10F, P20F, P30F, P40F, and P50F matrices and RF foam.

Foam	RF	P10F	P20F	P30F	P40F	P50F
Apparent density [kg/m^3^]	54.5	33.2	33.8	34.7	35.3	35.7
Porosity [%]	95.45	97.23	97.18	97.10	97.05	97.02

**Table 3:** Drug encapsulation efficiency and loading capacity calculations for PU–PAMAM matrices and RF foam.

Foam	RF	P10F	P20F	P30F	P40F	P50F
LC [%]	7.7	11.0	17.9	35.0	35.3	29.1
EE [%]	11	14	24	48	51	42

**Table 4:** Values of release exponents, *n*, and correlation coefficients, R^2^, for fitting experimental data using the Korsmeyer–Peppas model.

Foam	RF	P10F	P20F	P30F	P40F	P50F
*n* [-]	0.05	0.14	0.27	0.15	0.19	0.21
R^2^ [-]	0.998	0.989	0.997	0.997	0.861	0.984

## Data Availability

The original contributions presented in the study are included in the article. Further inquiries can be directed to the corresponding author.
